# 1,4-Dimethyl­piperazine-1,4-diium dibromide dihydrate

**DOI:** 10.1107/S1600536812020272

**Published:** 2012-05-12

**Authors:** Su-Wen Sun

**Affiliations:** a College of Chemistry and Chemical Engineering, Southeast University, Nanjing 211189, People’s Republic of China

## Abstract

In the title hydrated mol­ecular salt, C_6_H_16_N_2_^2+^·2Br^−^·2H_2_O, the complete 1,4-dimethyl­piperazine-1,4-diium dication is generated by crystallographic inversion symmetry and both exocyclic C—N bonds are in equatorial orientations. In the crystal, the components are linked by N—H⋯O and O—H⋯Br hydrogen bonds, generating chains propagating in [110].

## Related literature

For background to mol­ecular ferroelectrics, see: Fu *et al.* (2009 ▶). 
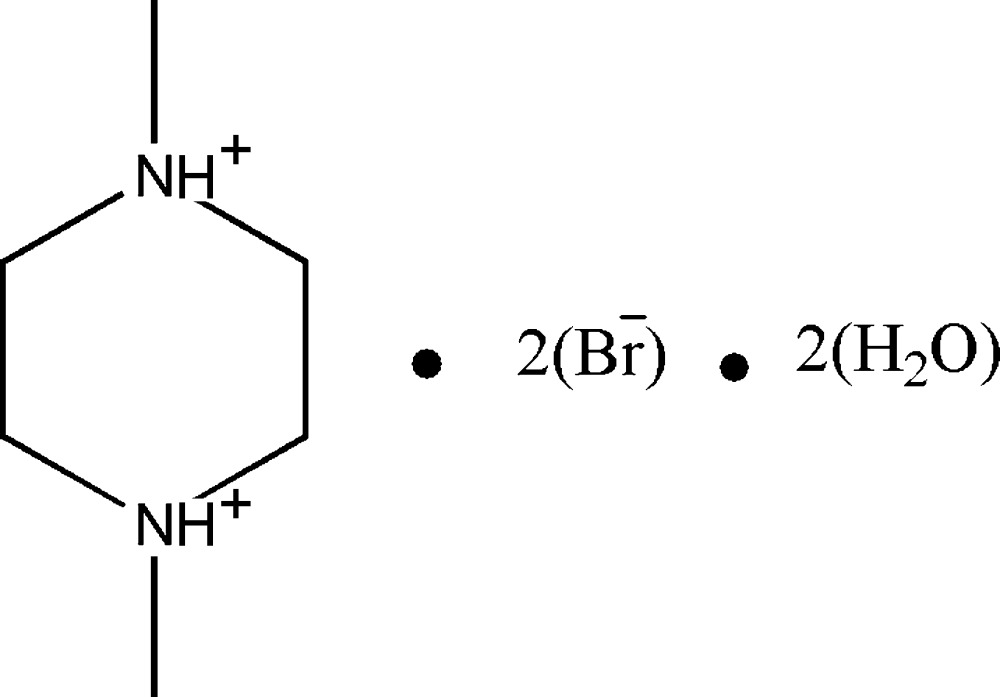


## Experimental

### 

#### Crystal data

C_6_H_16_N_2_^2+^·2Br^−^·2H_2_O
*M* *_r_* = 312.06
Triclinic, 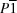

*a* = 6.2975 (13) Å
*b* = 7.0180 (14) Å
*c* = 7.2143 (14) Å
α = 71.54 (3)°
β = 86.62 (3)°
γ = 85.54 (3)°
*V* = 301.32 (10) Å^3^
*Z* = 1
Mo *K*α radiation
μ = 6.70 mm^−1^
*T* = 293 K
0.30 × 0.30 × 0.20 mm

#### Data collection

Rigaku SCXmini CCD diffractometer
Absorption correction: multi-scan (*CrystalClear*; Rigaku, 2005 ▶) *T*_min_ = 0.154, *T*_max_ = 0.262
3084 measured reflections
1370 independent reflections
1131 reflections with *I* > 2σ(*I*)
*R*_int_ = 0.067

#### Refinement

*R*[*F* ^2^ > 2σ(*F* ^2^)] = 0.059
*wR*(*F* ^2^) = 0.169
*S* = 1.02
1370 reflections
64 parameters
3 restraints
H atoms treated by a mixture of independent and constrained refinement
Δρ_max_ = 0.80 e Å^−3^
Δρ_min_ = −1.12 e Å^−3^

### 

Data collection: *CrystalClear* (Rigaku, 2005 ▶); cell refinement: *CrystalClear*; data reduction: *CrystalClear*; program(s) used to solve structure: *SHELXS97* (Sheldrick, 2008 ▶); program(s) used to refine structure: *SHELXL97* (Sheldrick, 2008 ▶); molecular graphics: *SHELXTL* (Sheldrick, 2008 ▶); software used to prepare material for publication: *SHELXL97*.

## Supplementary Material

Crystal structure: contains datablock(s) I, global. DOI: 10.1107/S1600536812020272/hb6759sup1.cif

Structure factors: contains datablock(s) I. DOI: 10.1107/S1600536812020272/hb6759Isup2.hkl

Supplementary material file. DOI: 10.1107/S1600536812020272/hb6759Isup3.cml

Additional supplementary materials:  crystallographic information; 3D view; checkCIF report

## Figures and Tables

**Table 1:** Hydrogen-bond geometry (Å, °)

*D*—H⋯*A*	*D*—H	H⋯*A*	*D*⋯*A*	*D*—H⋯*A*
N1—H1*A*⋯O1^i^	0.91	1.83	2.737 (9)	174
O1—H1*WA*⋯Br1^ii^	0.85 (6)	2.51 (9)	3.275 (7)	151 (11)
O1—H1*WB*⋯Br1	0.85 (3)	2.41 (4)	3.244 (7)	166 (7)

Symmetry codes: (i) 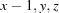; (ii) 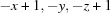.

**Fig. 1.**
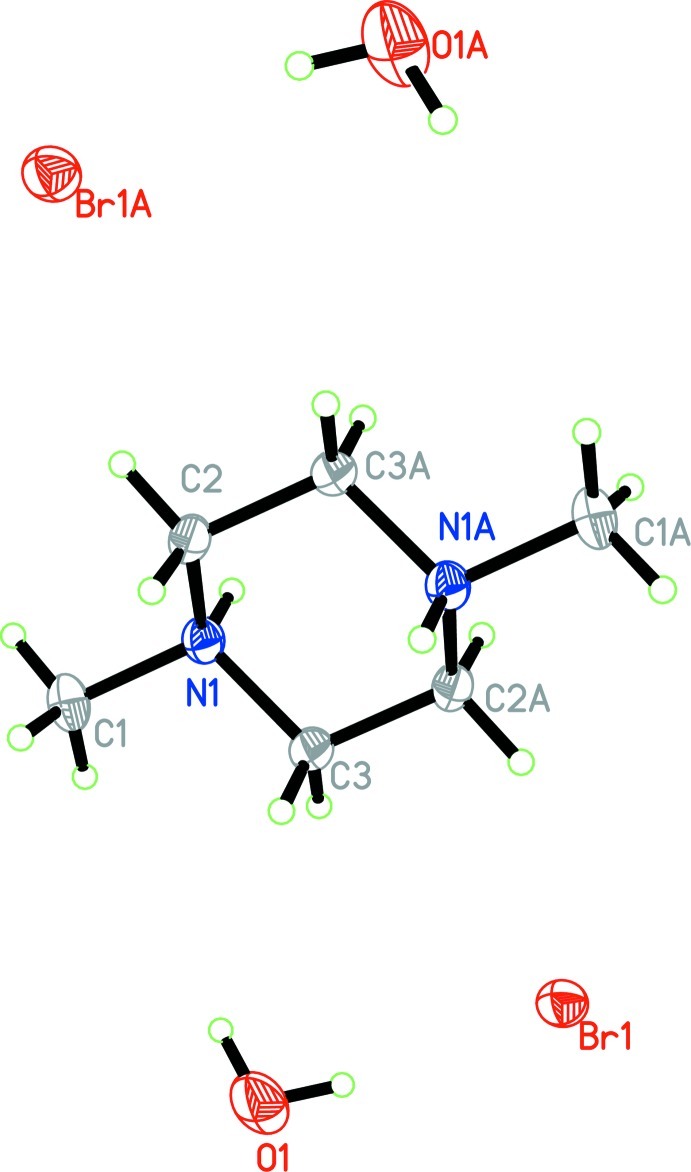
The molecular structure of the title compound with 30% probability displacement ellipsoids.

**Fig. 2.**
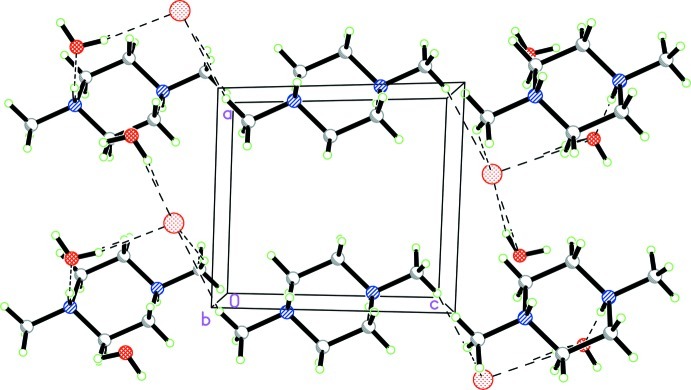
A view of the packing of the title compound, stacking along the b axis. Dashed lines indicate hydrogen bonds.

## supplementary crystallographic information

### Comment

Dielectric constant measurements of compounds as a function of temperature is the basic methods to find the materials which possess potential ferroelectric phase changes (Fu *et al.*, 2009). The dielectric constant of the title compound has been measured,but showed no dielectric disuniformity in the range 113–353 K(mp.403–410 K).

The asymmetric unit of the title compound is shown in Fig. 1. crystallized in the monoclinic P-1 space group, The crystal packing Fig. 2 features weak intermolecular O—H···Br and N—H···O hydrogen bonds (Table 1).

### Experimental

1,4-Dimethyl-piperazine (0.57 g) and an excess of hydrobromic acid (0.95 g) were dissolved in methanol without any precipitation under stirring at room temperature. Colourless blocks of the title compound were obtained by slow evaporation of a methanol solution at room temperature over two days.

### Refinement

H atoms were placed in calculated positions (N—H = 0.89 Å; C—H = 0.96 Å and 0.97 Å for C*sp^3^* atoms), assigned fixed *U*_iso_ values [1.5*U*eq(C*sp^3^*,*N*)] and allowed to ride.The H1WA and H1WB on the O1 were restrained with O—H =0.85 Å yielding O1—H1 = 0.8448 Å and O1 —H2 = 0.8440 Å, with *U*_iso_(H) = 1.2 *U*_iso_(O).

### Crystal data

**Table:**

C_6_H_16_N_2_^2+^·2Br^−^·2H_2_O	*V* = 301.32 (10) Å^3^
*M**_r_* = 312.06	*Z* = 1
Triclinic, *P*1	*F*(000) = 156
Hall symbol: -P 1	*D*_x_ = 1.720 Mg m^−^^3^
*a* = 6.2975 (13) Å	Mo *K*α radiation, λ = 0.71073 Å
*b* = 7.0180 (14) Å	θ = 3.1–27.5°
*c* = 7.2143 (14) Å	µ = 6.70 mm^−^^1^
α = 71.54 (3)°	*T* = 293 K
β = 86.62 (3)°	Block, colorless
γ = 85.54 (3)°	0.30 × 0.30 × 0.20 mm

### Data collection

**Table:**

Rigaku SCXmini CCD diffractometer	1370 independent reflections
Radiation source: fine-focus sealed tube	1131 reflections with *I* > 2σ(*I*)
Graphite monochromator	*R*_int_ = 0.067
Detector resolution: 13.6612 pixels mm^-1^	θ_max_ = 27.5°, θ_min_ = 3.1°
CCD_Profile_fitting scans	*h* = −8→7
Absorption correction: multi-scan (*CrystalClear*; Rigaku, 2005)	*k* = −9→9
*T*_min_ = 0.154, *T*_max_ = 0.262	*l* = −9→9
3084 measured reflections	

### Refinement

**Table:**

Refinement on *F*^2^	Primary atom site location: structure-invariant direct methods
Least-squares matrix: full	Secondary atom site location: difference Fourier map
*R*[*F*^2^ > 2σ(*F*^2^)] = 0.059	Hydrogen site location: inferred from neighbouring sites
*wR*(*F*^2^) = 0.169	H atoms treated by a mixture of independent and constrained refinement
*S* = 1.02	*w* = 1/[σ^2^(*F*_o_^2^) + (0.0897*P*)^2^ + 0.3362*P*] where *P* = (*F*_o_^2^ + 2*F*_c_^2^)/3
1370 reflections	(Δ/σ)_max_ = 0.024
64 parameters	Δρ_max_ = 0.80 e Å^−^^3^
3 restraints	Δρ_min_ = −1.12 e Å^−^^3^

### Special details

**Table:**

Geometry. All e.s.d.'s (except the e.s.d. in the dihedral angle between two l.s. planes) are estimated using the full covariance matrix. The cell e.s.d.'s are taken into account individually in the estimation of e.s.d.'s in distances, angles and torsion angles; correlations between e.s.d.'s in cell parameters are only used when they are defined by crystal symmetry. An approximate (isotropic) treatment of cell e.s.d.'s is used for estimating e.s.d.'s involving l.s. planes.
Refinement. Refinement of *F*^2^ against ALL reflections. The weighted *R*-factor *wR* and goodness of fit *S* are based on *F*^2^, conventional *R*-factors *R* are based on *F*, with *F* set to zero for negative *F*^2^. The threshold expression of *F*^2^ > σ(*F*^2^) is used only for calculating *R*-factors(gt) *etc*. and is not relevant to the choice of reflections for refinement. *R*-factors based on *F*^2^ are statistically about twice as large as those based on *F*, and *R*- factors based on ALL data will be even larger.

### Fractional atomic coordinates and isotropic or equivalent isotropic displacement parameters (Å^2^)

**Table:**

	*x*	*y*	*z*	*U*_iso_*/*U*_eq_	
N1	0.0403 (8)	0.3473 (7)	0.6882 (7)	0.0319 (11)	
H1A	−0.0362	0.2477	0.6754	0.038*	
C2	−0.1103 (10)	0.5311 (10)	0.6709 (9)	0.0355 (13)	
H2A	−0.2225	0.4973	0.7717	0.043*	
H2B	−0.0331	0.6371	0.6904	0.043*	
C3	0.2067 (9)	0.3955 (10)	0.5259 (9)	0.0346 (13)	
H3A	0.2991	0.2759	0.5350	0.042*	
H3B	0.2934	0.4979	0.5406	0.042*	
C1	0.1381 (11)	0.2748 (10)	0.8819 (10)	0.0428 (15)	
H1B	0.2564	0.1808	0.8788	0.064*	
H1C	0.0342	0.2098	0.9796	0.064*	
H1D	0.1872	0.3869	0.9129	0.064*	
Br1	0.61718 (10)	0.21220 (10)	0.19573 (10)	0.0445 (3)	
O1	0.7864 (11)	0.0695 (11)	0.6378 (10)	0.0737 (19)	
H1WA	0.675 (9)	0.027 (18)	0.707 (10)	0.11 (5)*	
H1WB	0.757 (12)	0.090 (12)	0.519 (4)	0.05 (2)*	

### Atomic displacement parameters (Å^2^)

**Table:**

	*U*^11^	*U*^22^	*U*^33^	*U*^12^	*U*^13^	*U*^23^
N1	0.035 (3)	0.031 (2)	0.028 (3)	−0.003 (2)	−0.002 (2)	−0.007 (2)
C2	0.037 (3)	0.043 (3)	0.027 (3)	0.001 (3)	0.002 (2)	−0.012 (3)
C3	0.031 (3)	0.041 (3)	0.031 (3)	0.003 (2)	−0.001 (2)	−0.013 (3)
C1	0.056 (4)	0.043 (4)	0.028 (3)	0.000 (3)	−0.010 (3)	−0.008 (3)
Br1	0.0393 (4)	0.0481 (5)	0.0451 (5)	−0.0090 (3)	−0.0021 (3)	−0.0116 (3)
O1	0.080 (4)	0.084 (4)	0.055 (4)	−0.048 (4)	−0.012 (3)	−0.007 (3)

### Geometric parameters (Å, º)

**Table:**

N1—C1	1.482 (8)	C3—H3A	0.9700
N1—C3	1.498 (8)	C3—H3B	0.9700
N1—C2	1.516 (8)	C1—H1B	0.9600
N1—H1A	0.9100	C1—H1C	0.9600
C2—C3^i^	1.497 (9)	C1—H1D	0.9600
C2—H2A	0.9700	O1—H1WA	0.853 (10)
C2—H2B	0.9700	O1—H1WB	0.854 (10)
C3—C2^i^	1.497 (9)		
			
C1—N1—C3	111.3 (5)	N1—C3—H3A	109.2
C1—N1—C2	111.1 (5)	C2^i^—C3—H3A	109.2
C3—N1—C2	109.7 (5)	N1—C3—H3B	109.2
C1—N1—H1A	108.2	C2^i^—C3—H3B	109.2
C3—N1—H1A	108.2	H3A—C3—H3B	107.9
C2—N1—H1A	108.2	N1—C1—H1B	109.5
C3^i^—C2—N1	110.7 (5)	N1—C1—H1C	109.5
C3^i^—C2—H2A	109.5	H1B—C1—H1C	109.5
N1—C2—H2A	109.5	N1—C1—H1D	109.5
C3^i^—C2—H2B	109.5	H1B—C1—H1D	109.5
N1—C2—H2B	109.5	H1C—C1—H1D	109.5
H2A—C2—H2B	108.1	H1WA—O1—H1WB	107 (3)
N1—C3—C2^i^	111.9 (5)		
			
C1—N1—C2—C3^i^	179.4 (5)	C1—N1—C3—C2^i^	180.0 (5)
C3—N1—C2—C3^i^	56.0 (7)	C2—N1—C3—C2^i^	−56.7 (7)

Symmetry code: (i) −*x*, −*y*+1, −*z*+1.

### Hydrogen-bond geometry (Å, º)

**Table:**

*D*—H···*A*	*D*—H	H···*A*	*D*···*A*	*D*—H···*A*
N1—H1*A*···O1^ii^	0.91	1.83	2.737 (9)	174
O1—H1*WA*···Br1^iii^	0.85 (6)	2.51 (9)	3.275 (7)	151 (11)
O1—H1*WB*···Br1	0.85 (3)	2.41 (4)	3.244 (7)	166 (7)

Symmetry codes: (ii) *x*−1, *y*, *z*; (iii) −*x*+1, −*y*, −*z*+1.
